# Effects of plant population density and root-induced cytokinin on the corn compensatory growth during post-drought rewatering

**DOI:** 10.1371/journal.pone.0198878

**Published:** 2018-06-28

**Authors:** Xiao-Ling Wang, Rong-Rong Qin, Run-Hong Sun, Xiao-Gai Hou, Lin Qi, Jiang Shi

**Affiliations:** 1 Department of biological sciences, College of Agronomy, Henan University of Science and Technology, Luoyang, Henan Province, China; 2 Institute of Plant Protection Research, Henan Academy of Agricultural Sciences, Henan Key Laboratory for Control of Crop Diseases and Insect Pests, IPM Key Laboratory in Southern Part of North China for Ministry of Agriculture, Zhengzhou, Henan Province, China; 3 Department of biotechnology, College of Agronomy, Henan University of Science and Technology, Luoyang, Henan Province, China; Beijing Forestry University, CHINA

## Abstract

The effect of plant population density (PPD) and root-induced leaf cytokinin on the compensatory growth of potted corn seedlings during post-drought rewatering was investigated. The study design comprised four treatments: (1) wetness with low PPD, (2) wetness with high PPD, (3) rewatering with low PPD, and (4) rewatering with high PPD. Results showed that drought stress restrained the growth of corns. By contrast, rewatering enhanced the net photosynthetic rate and growth of corns. During the 8 days of rewatering, compensatory growth during post-drought rewatering occurred in corns with high PPD; however, such compensatory growth did not occur in corns with low PPD. Zeatin riboside concentrations in leaves and xylem saps were significantly higher under rewatering treatment than those under wet treatment. High leaf cytokinin concentration accelerated corn growth. The coefficients of variation and Gini-coefficient of wet treatment were significantly higher than those of rewatering treatment under high PPD, demonstrating that intense intraspecific competition occurred in the wet treatment. Extreme intraspecific competition negatively affected net photosynthetic rate. In brief, the interactions between root-induced leaf cytokinin and weak intraspecific competition promoted the compensatory growth under high PPD.

## Introduction

Plant compensatory growth theory during post-drought rewatering underlies the extensive application of water-saving agriculture techniques, which mostly involve regulated deficit irrigation, deficit irrigation technologies, and dryland agriculture [1–5]. Compensatory growth is defined as the increase in plant growth rate in response to water availability after drought. The mechanism of compensatory growth during post-drought rewatering has been investigated in numerous crops, including maize, wheat, Kentucky bluegrass, and cotton [6–9]. Photosynthetic rate, stomatal conductance, fertiliser use, anti-aging properties, and other attributes show close association with compensatory growth. Crop production is essentially controlled by plant population density (PPD). However, the population-level mechanism underlying crop compensatory growth during post-drought rewatering remains poorly understood and thus warrants further investigation.

PPD continues to receive considerable research attention given that it is the basic attribute of crop populations. Rossini et al. [10] found that corn (*Zea mays* L.) production is drastically affected by the combination of high PPD and water stress. Peake et al. [11] used maize simulation models to determine the optimum PPD for rain-fed environments. The effects of deficit irrigation and PPD on cotton production have been studied to reveal the key factors that affect cotton yield under deficit irrigation [12]. Thus, PPD is closely associated with crop water use and drought resistant. Investigation of the effect of PPD on crop compensatory growth during post-drought rewatering can possibly provide a new idea for this mechanism.

Corn compensatory growth during post-drought rewatering essentially involves rapid growth under the stimulus of sufficient water supply. This stimulus is directly sensed by the roots. Wang et al. [13] found that the roots contribute the compensatory growth of corn during post-drought rewatering by inducing leaf cytokinin. PPD plays an important role in root functions by affecting plant growth. Therefore, the contributions of PPD and root-induced leaf cytokinin to compensatory growth during post-drought rewatering must be investigated.

The present study investigated the compensatory growth of corn (*Zea mays* L.) at the seedling stage during post-drought rewatering, Corn, China’s principal crop and the world’s third most popular crop, is highly water consuming. Corn growth and productivity in Northern China are usually restrained by water shortage. Thus, water-use efficiency must be increased to improve corn production in the region. In the study, we aimed to determine the effect of PPD on corn compensatory growth of post-drought rewatering on the basis of root-induced leaf cytokinin. To achieve this object, we investigated the effect of intensity of intraspecific competition and root-induced leaf cytokinin on the photosynthetic rate of corns planted at different PPDs. We determined the leaf gibberellic acid (GA_3_), abscisic acid (ABA), indole-3-acetic acid (IAA) and zeatin riboside (ZR) contents, and ZR and ABA contents in xylem saps under different treatments. We also determined the net photosynthetic rate (P_n_), stomatal conductance (G_s_), transpiration rate (T_r_), coefficients of variation (CV), and the Gini coefficient (*G*) of corn plants under different treatments.

## Materials and methods

### Experimental design

The study was conducted in the experimental farm of Henan University of Science and Technology. The farm (34°32' N, 112°16' E and altitude 138 m) is located in Luoyang City of Henan Province and has a warm temperate continental monsoon climate. The annual rainfall and temperature of the farm are 601 mm and 14.2°C, respectively. The corn cultivar ‘Zhendan-958’, which is widely planted in China, was used because of its drought resistance and wide adaptability. Corn plants at the seedling stage were used as the test material because of their rapid growth and easily detectable variations in growth parameters. The study was conducted under a rain shelter in pots filled with sand. Sand was employed as the substrate to facilitate root separation and rinsing.

**Fig 1:**
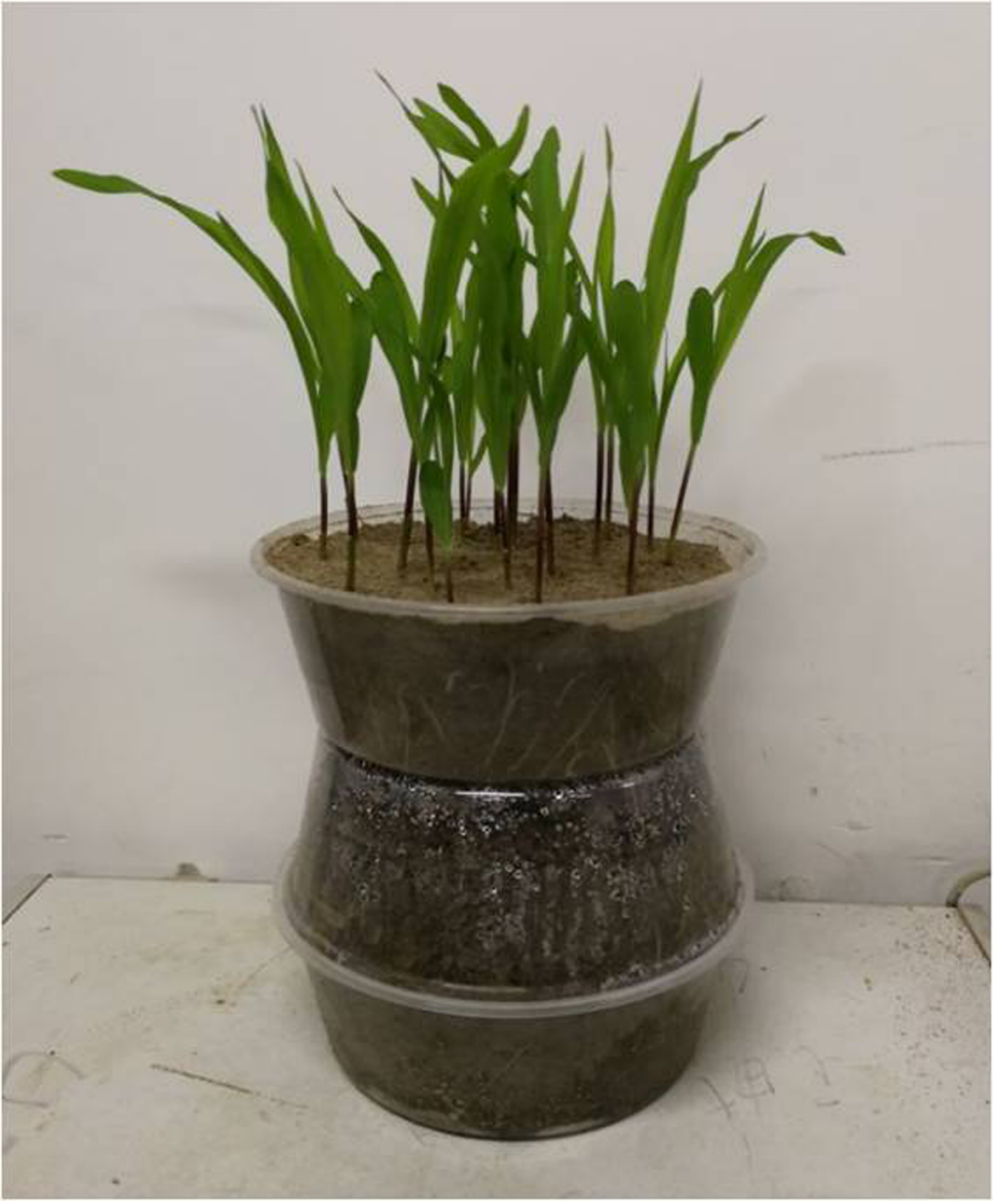
Three single pots stacked together. A, B, and C refer to top pot, middle pot, and bottom pot, respectively.

Corn seedlings were planted in three single plastic pots stacked together (Fig 1). This pot configuration was adopted to provide sufficient space for planting corn seedlings at different PPDs. The mouth diameter, base diameter, and height of each single pot was 23.5, 19.5, and 8.8 cm, respectively. As shown in Fig 1, the base of the middle and top pots were removed, and the middle pot was inverted and stacked on the bottom pot. The mouth of the pots were held together with transparent adhesive tape. Similarly, the top pot was stacked on the middle pot, and the bottoms of the pots were held together with transparent adhesive tape. The bases of the bottom pot were perforated to allow water to flow through. To ensure that the seedlings grew normally, the seedlings were watered daily with modified Hoagland solution, containing 5 mM K, 8 mM Ca, 1 mM P, 1 mM Mg, 89 μM Fe, 18 μM Mn, 0.9 μM Cu, 1.75 μM Zn, and 15 mM NO_3_^−^.

In May, 2017, 200 stacked pots were prepared for the experiment. The experiment was conducted from May 8, 2017 to June 16, 2017 for a total of 40 days. On 8 May, 2017, 24 corn seeds were sown in each stacked pot. Approximately 6 days after seedling emergence, pots containing 4 and 12 seedlings exhibiting vigorous growth were allocated to the low and high PPD groups, respectively, and the other seedlings were thinned out. After 16 days of growth, 36 pots with uniformly grown seedlings were selected. A total of 18 pots were allocated to each PPD treatment, and the entire study population comprised 288 corn seedlings.

The 18 pots for each PPD were divided into two groups. Each group comprised nine pots. On the basis of preliminary experiments, 10 days of drought stress could positively affect corn growth during post-drought rewatering. Furthermore, the variations in growth parameters during 8 days of post-drought rewatering under high and low PPDs could be easily detected. Therefore, two growth periods were established to detect compensatory growth during post-drought rewatering under different PPDs. The growth periods involved 10 days of drought stress and 8 days of rewatering. During the drought stress period, one group under each PPD treatment received sufficient water supply, and the other group was subjected to drought stress. All groups received sufficient water supply during the rewatering period. Thus, the experiment involved four treatments: (1) wetness with low PPD (WL), (2) wetness with high PPD (WH), (3) rewatering with low PPD (DL), (4) rewatering with high PPD (DH). No corn plant died under any treatment during the experimental period. Each treatment group included nine pots.

Wetness and drought stress were defined as the soil water content (SWC) of 75%–80% and 45%–50% of the field water capacity (FWC), respectively. SWC was determined by weighing the pots every 5 h over the period of 6:00 am to 9:00 pm. When the SWC decrease below 75% of the FWC, water was added to maintain the water content at 75%–80% of the FWC. For inducing drought stress, water was not added to the soil during the first 2–3 days of treatment to allow the soil water to dissipate. Subsequently, the pots were weighed, and water was added to maintain the SWC at 45%–50% of FWC. In accordance with Xiong et al. [14], the SWC at each treatment hour was calculated using the following formula (1):

$$SWC=\frac{W_{t}-W_{d}-W_{e}-W_{p}}{W_{d}\times FWC}\times 100\%$$ (1)

where *W*_*t*_ is the temporary whole pot weight, *W*_*d*_ is the net weight of the dried sand in the pot, *W*_*e*_ is the weight of the empty pot and *W*_*p*_ is the estimated fresh weight (FW) of all plants in the pot and FWC is the field water capacity. The estimated FW of all plants in one pot for ech test period was determined in advance by using extra pots.

P_n_, G_s_, and T_r_ in each treatment were measured at the end of the drought-stress period. At the end of the drought-stress period, three pots from each treatment were transported to the laboratory for the measurement of root, stem and leaf biomasses; IAA, GA_3_,ABA, and ZR contents in leaves; ZR and ABA contents in xylem saps; soluble carbohydrates content in roots; CV and *G*. All indexes measured during the drought-stress period were tested again during the rewatering period. P_n_, G_s_, and T_r_ of each treatment were measured at 2, 4, 6, and 8 days after rewatering. At 4 and 8 days after rewatering, three pots from each treatment were also transported to the laboratory to evaluate biomass, and plant hormones, soluble carbohydrates content in roots, CV, and *G*.

### Measurements and data analysis

#### Biomass, photosynthetic rate and size inequality

Corn roots were separated from soil through washing. Fresh root, stem, and leaf samples were dried for 72 h in a forced air oven at 65°C and used for biomass determination. The water-soluble carbohydrates contents of corn roots were measured through the anthrone colorimetric method [15]. LI-6400 photosynthesis equipment was used to determine P_n_, G_s_, and T_r_ at 11:00 everyday. The amount of extracted xylem sap was measured through weighing as follows. The stem bases of seedlings were wounded through clipping. The wounds were immediately covered with approximately 0.2 g of absorbent cotton. The cotton was then tightly wrapped with plastic sheeting to prevent water evaporation. The cotton piece was reweighed after 12 h. The increase in the weight of the absorbent cotton piece was considered the amount of xylem sap collected from the roots. Sap volume was determined by dividing the increase in weight by 1 g/cm^3^. The cotton piece was placed and compacted in the bottom of a 10 mL syringe with a piston. The filtrate was collected into a 5 ml centrifuge tube. About 1 ml of distilled water was added to the cotton to extract the absorbed xylem sap, and the cotton was compressed with the piston. This process was repeated twice. Extracts were pooled in the same 5 ml centrifuge tube. The collected xylem sap was used for the measurement of ZR and ABA contents.

The degree of size inequality for each treatment was estimated on the basis of CV and *G* [16–20].

*G* is given by formula (2):

$$G=\frac{\sum_{i=1}^{n}\sum_{j=1}^{n}\left|x_{i}-x_{j}\right|}{2n^{2}\bar{x}}$$ (2)

where *x*_*i*_ and *x*_*j*_ are the sizes of individuals *i* and *j*, respectively. Individual size was indicated as aboveground biomass.

#### Hormones

After xylem sap extraction, all of the xylem sap collected in the 5 ml centrifuge tube was immediately injected into a solid-phase extraction C-18 column (Waters Corporation, USA), blow-dried with nitrogen and then stored at−80°C for the future measurement of hormone concentrations. Fresh leaf samples (1–3 g) were frozen for 30 min in liquid nitrogen and then stored at −80°C for the same purpose. Each frozen leaf sample was cut into several pieces, and approximately 0.7 g of the leaf sample was mixed with 80% methanol containing 1 mmol/L di-tert-butyl-4-methylphenol. The mixture was ground into a homogenate in a water bath and was extracted at 4°C for 4 h. The samples were centrifuged at 7,000 rpm for 15 min and then precipitated, and the supernatant was collected. The precipitate was extracted with 80% methanol for 1 h, and the supernatant was again collected. The supernatants were pooled, injected into a solid-phase extraction C-18 column and blow-dried with nitrogen. The residues of leaf and xylem sap extract samples were dissolved in 0.01 mol/L phosphate buffer solution (pH 7.4) and subjected to enzyme-linked immunosorbent assay (ELISA) to determine IAA, GA_3_, ABA, and ZR concentrations in accordance with previously described methods [21–23].

The Mouse monoclonal antigens and antibodies against IAA, GA_3_, ABA and ZR, and immunoglobulin G (IgG)–horseradish peroxidase used in ELISA were produced at the Phytohormone Research Institute of China Agricultural University. IAA, GA_3_, ABA and ZR were quantified using the hormone test kit produced by the institute, as described in previous plant hormonal studies [21–23]. ELISA was performed in a 96-well microtitration plate. Each well was coated with 100 μL of coating buffer (1.5 g/L Na_2_CO_3_, 2.93 g/L NaHCO_3_ and 0.02 g/L NaN_3_ at pH 9.6) containing 0.25 μg/mL hormone antigens. After washing four times with phosphate-buffered saline (PBS) + Tween 20 [0.1% (v/v)] buffer (pH 7.4), each well was filled with 50 μL of either corn sample extract or IAA, GA_3_, ABA, and ZR standards and 50 μL of 20 μg/mL antibodies against IAA, GA_3_, ABA, and ZR.

The plates were incubated for 3 h at 28°C to determine GA_3_, ABA, and ZR concentrations and overnight at 4°C to determine the IAA concentration. Subsequently, the plates were washed for four times with PBS + Tween 20 [0.1% (v/v)] buffer (pH 7.4). Next, 100 μL of 1.25 μg/mL IgG–horseradish peroxidase substrate was added to each well, and the plates were incubated for 1 h at 30°C. The plates were rinsed for five times with PBS + Tween 20 buffer, and 100 μL of color-appearing solution containing 1.5 mg mL^−1^ *o*-phenylenediamine and 0.008% (v/v) H_2_O_2_ were added to each well. When the highest concentration and 0 ng/mL of the standard exhibited pale and deep colors, respectively, the reaction was terminated by adding 50 μL of 6 N H_2_SO_4_ per well. Color development in each well was detected using an ELISA Reader (Model DG-5023, Huadong Electron Tube Factory, Nanjing, China) at an optical density of A_490_.

On the basis of machine-readable optical density, the concentrations of IAA, GA_3_, ABA, and ZR dissolved in the buffer solution were calculated. The amounts of IAA, GA_3_, ABA, and ZR were obtained by multiplying their concentrations in buffer solution and the buffer solution volumes. The amounts of IAA, GA_3_, ABA, and ZR were divided by their leaf sample weight to obtain their contents in leaf, and then divided by the volume of xylem sap to obtain the ZR and ABA contents in xylem sap. The percentage recoveries of IAA, GA_3_, ABA, and ZR, which were determined by adding known quantities of the standard hormone to a split extract, were 79.2%, 78.6%, 80.2%, and 83.0%, respectively. These results indicate the absence of nonspecific inhibitors in the extracts. Specificity and other possible nonspecific immunoreactive interference of monoclonal antibodies used were previously examined by He [24]. Prior to xylem sap extraction, absorbent cotton was soaked in methanol and air dried three times to reduce contamination in ABA and ZR measurements.

### Statistical analysis

The abbreviations used in the text are listed in Table 1. Microsoft Excel 2007 was used for statistical analysis, and values in the tables or graphs represent averages. The effects of PPD and rewatering on biomass, leaf IAA, GA_3_, ABA, and ZR contents, ZR and ABA contents in xylem saps, solute carbohydrate content in roots, P_n_, G_s_, and T_r_ were analyzed using multiple Duncan range tests (P = 0.05). Linear regression was used to evaluate the relationship between leaf ZR content and ZR content in xylem sap.

**Table 1:** Symbol definition.

Symbol	Definition	Symbol	Definition
PPD	Plant population density	W_e_	Weight of empty pot
GA_3_	Gibberellic acid	W_p_	Estimated fresh weight
ABA	Abscisic acid	W_t_	Temporary whole pot weight
IAA	Indole-3-acetic acid	W_d_	Net weight of dried sand in the pot
ZR	Zeatin riboside	WL	Wetness treatment with low PPD
P_n_	Net photosynthetic rate	WH	Wetness treatment with high PPD
T_r_	Transpiration rate	DL	Rewatering treatment with low PPD
G_s_	Stomatal conductance	DH	Rewatering treatment with high PPD
ELISA	Enzyme-linked immunosorbent assay	CV	Coefficients of variation
FW	Fresh weight	G	Gini-coefficient
FWC	Field water capacity	x_i_	the size of individuals i
SWC	Soil water content	x_j_	the size of individuals j

## Result

### Biomass

As showed in Table 2, the aboveground biomass and total biomasses per pot were significantly higher in WL than in DL and in WH than in DH before rewatering, thereby indicating that drought stress inhibited corn growth. However, rewatering enhanced corn growth. At 8 days after rewatering, no significant difference in the aboveground and total biomasses per pot was noted between WH and DH. Although WL showed significantly higher aboveground and total biomasses per pot at 8 days after rewatering compared with DL, rewatering still increased corn growth in DL. From before rewatering to 8 days after rewatering, the aboveground and total biomasses per pot in DL increased 1.55 and 1.49 times, respectively. In general, when the reduced biomass of rewatering corns caused by drought stress is compensated or exceeded during the rewatering period, compensatory growth occurs. Therefore, compensatory growth occurred in corns with high PPD during the 8 days of rewatering.

**Table 2:** Biomasses of corn in the different treatments.

Treatments	Days after rewatering			Days after rewatering		
	0	4	8	0	4	8
	Aboveground biomass per pot (g/pot)			Aboveground biomass per plant g/plant)		
WL	2.36±0.14c	2.74±0.18c	2.79±0.09b	0.59±0.04a	0.69±0.05a	0.70±0.02a
WH	4.20±0.18a	4.52±0.27a	4.72±0.10a	0.35±0.02c	0.38±0.02c	0.39±0.01c
DL	1.64±0.14d	2.32±0.12d	2.55±0.06c	0.41±0.04b	0.58±0.03b	0.64±0.02b
DH	3.17±0.14b	4.08±0.24b	4.66±0.20a	0.26±0.01d	0.34±0.02d	0.39±0.02c
	Total biomass per pot(g/pot)			Total biomass per plant (g/plant)		
WL	3.85±0.17c	4.53±0.15c	4.71±0.16b	0.96±0.04a	1.13±0.04a	1.18±0.04a
WH	6.96±0.32a	7.56±0.35a	7.93±0.20a	0.58±0.03c	0.63±0.03c	0.66±0.02c
DL	2.86±0.23d	3.95±0.20d	4.26±0.15c	0.72±0.06b	0.99±0.05b	1.07±0.04b
DH	5.58±0.23b	7.06±0.30b	7.84±0.31a	0.47±0.02d	0.59±0.03d	0.65±0.03c
	Root biomass per pot (g/pot)			Root biomass per plant (g/plant)		
WL	1.49±0.05c	1.79±0.06b	1.91±0.07b	0.37±0.01a	0.45±0.02a	0.48±0.02a
WH	2.76±0.18a	3.05±0.10a	3.21±0.11a	0.23±0.02c	0.25±0.01c	0.27±0.01c
DL	1.22±0.09d	1.63±0.09c	1.71±0.09c	0.31±0.02b	0.41±0.02b	0.43±0.02b
DH	2.42±0.09b	2.98±0.09a	3.18±0.14a	0.20±0.01d	0.25±0.01c	0.27±0.01c
	Root-shoot ratio (%)					
WL	63.12±3.13b	65.33±5.83b	68.54±0.27a			
WH	65.81±3.52b	67.53±3.13ab	68.17±0.90a			
DL	74.13±1.46a	70.12±1.00ab	67.23±1.73a			
DH	76.24±1.24a	73.32±3.75a	68.17±2.60a			

The values are the mean± 1 standard deviation (n = 3), and for each number of days after rewatering, the different letters in each column indicate significant differences (P ≤ 0.05). WL: wetness with low PPD; WH: wetness with high PPD; DL: rewatering with low PPD; DH: rewatering with high PPD.

Before rewatering and at 4 and 8 days after rewatering, WL and DL showed significantly higher aboveground and total biomasses per plant than WH and DH, respectively. These results indicated that high PPD is disadvantageous for corn growth under wet conditions, drought stress, or rewatering, mainly because reduced growth in corn is due to the severe intraspecific competition in corn population under high PPD. Significantly higher root–shoot ratio was observed in DL and DH than in WL and WH before rewatering, but no significant difference was observed among treatment groups at 4 and 8 days after rewatering. Therefore, drought stress induced the occurrence of relatively large roots in corn seedlings, and rewatering decreased root size.

### Photosynthetic characteristics

**Fig 2:**
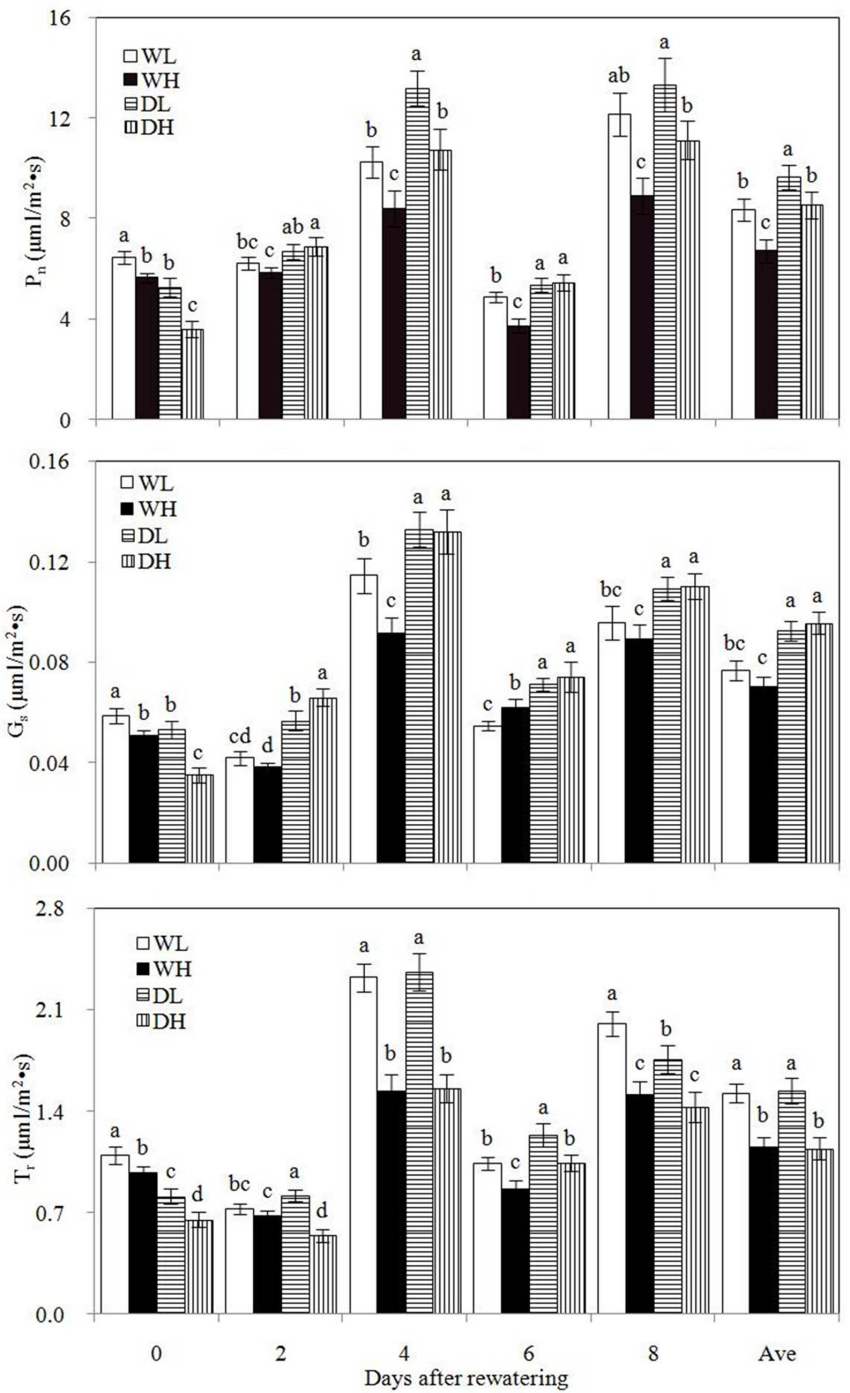
Net photosynthetic rate (P_n_), stomatal conductance (G_s_), and transpiration rate (T_r_) in the different treatment groups. “Ave” means the average value during the rewatering period. Error bars indicate ± 1 standard deviation. For each number of days after rewatering, the different letters indicate significant differences at P ≤ 0.05.WL: wetness with low PPD; WH: wetness with high PPD; DL: rewatering with low PPD; DH: rewatering with high PPD.

WL and WH showed significantly higher P_n_, G_s_, and T_r_ compared with DL and DH, respectively, before rewatering (Fig 2), demonstrating that drought stress restrained photosynthesis. During the rewatering period, DL and DH showed significantly higher average P_n_ and G_s_ than WL and WH, respectively. This finding showed that rewatering could increase P_n_ and G_s_. However, during the rewatering period, DL and DH showed 15.31% and 27.20% higher average P_n_ than WL and WH, respectively. Thus, under high PPD, rewatering showed increased tendency to cause a relatively large increase in the leaf photosynthetic rate. The same horizontal concentration for *T*_*r*_ was observed between WL and DL and between WH and DH during rewatering.

During the drought stress and rewatering, significantly higher P_n_, G_s_, and T_r_ occurred in WL than in WH, and significantly higher P_n_, G_s_, and T_r_ were observed in DL than in DH during drought stress period. These results indicated that high PPD decreased P_n_, G_s_, and T_r_ of corn under wet or drought stress. No significant increase in P_n_, G_s_, and T_r_ was observed in DL relative to DH during rewatering. Therefore, under rewatering conditions, high plant density did not decrease the photosynthetic indexes.

### Size hierarchy and soluble carbohydrate

**Fig 3:**
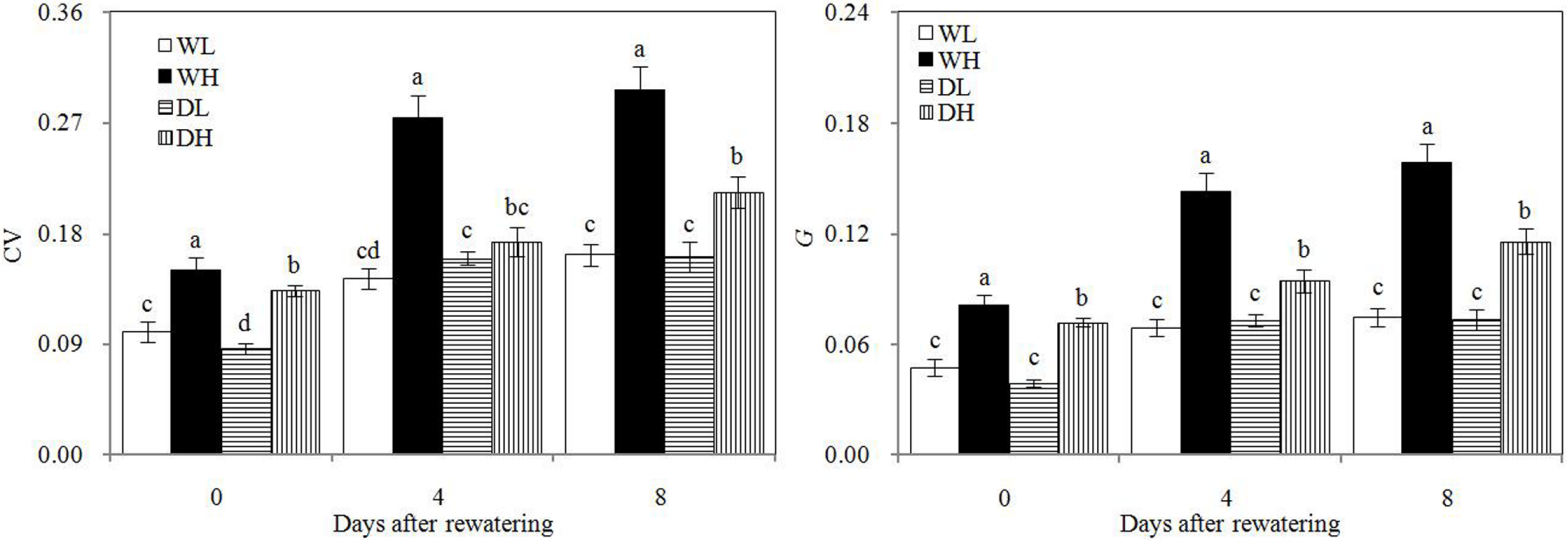
Coefficients of variation (*CV*) and the Gini coefficient (*G*) in different treatment groups. Error bars indicate ± 1 standard deviation. For each number of days after rewatering, the different letters indicate significant differences at P ≤ 0.05.WL: wetness with low PPD; WH: wetness with high PPD; DL: rewatering with low PPD; DH: rewatering with high PPD.

As showed in Fig 3, CV and *G* in all treatments increased before rewatering to 8 days after rewatering. CV and *G* are indexes that show population size hierarchy, and they measure the intensity of intraspecific competition. Our findings showed that the intensity of intraspecific competition increased with corn growth mainly because corn plants compete for more light, water, or soil nutrients as they grow larger. WH and DH showed significantly higher CV and *G* than WL and DL, respectively, at 4 and 8 days after rewatering. Therefore, intense intraspecific competition occurred in corn population with high PPD under wet conditions or under rewatering. WH showed significantly higher CV and *G* than DH at 4 and 8 days after rewatering, thereby demonstrating intense intraspecific competition in the wet corn population under high PPD. However, this phenomenon did not occur in the corn population under low PPD because of sufficient growth resources for the corn plants.

**Fig 4:**
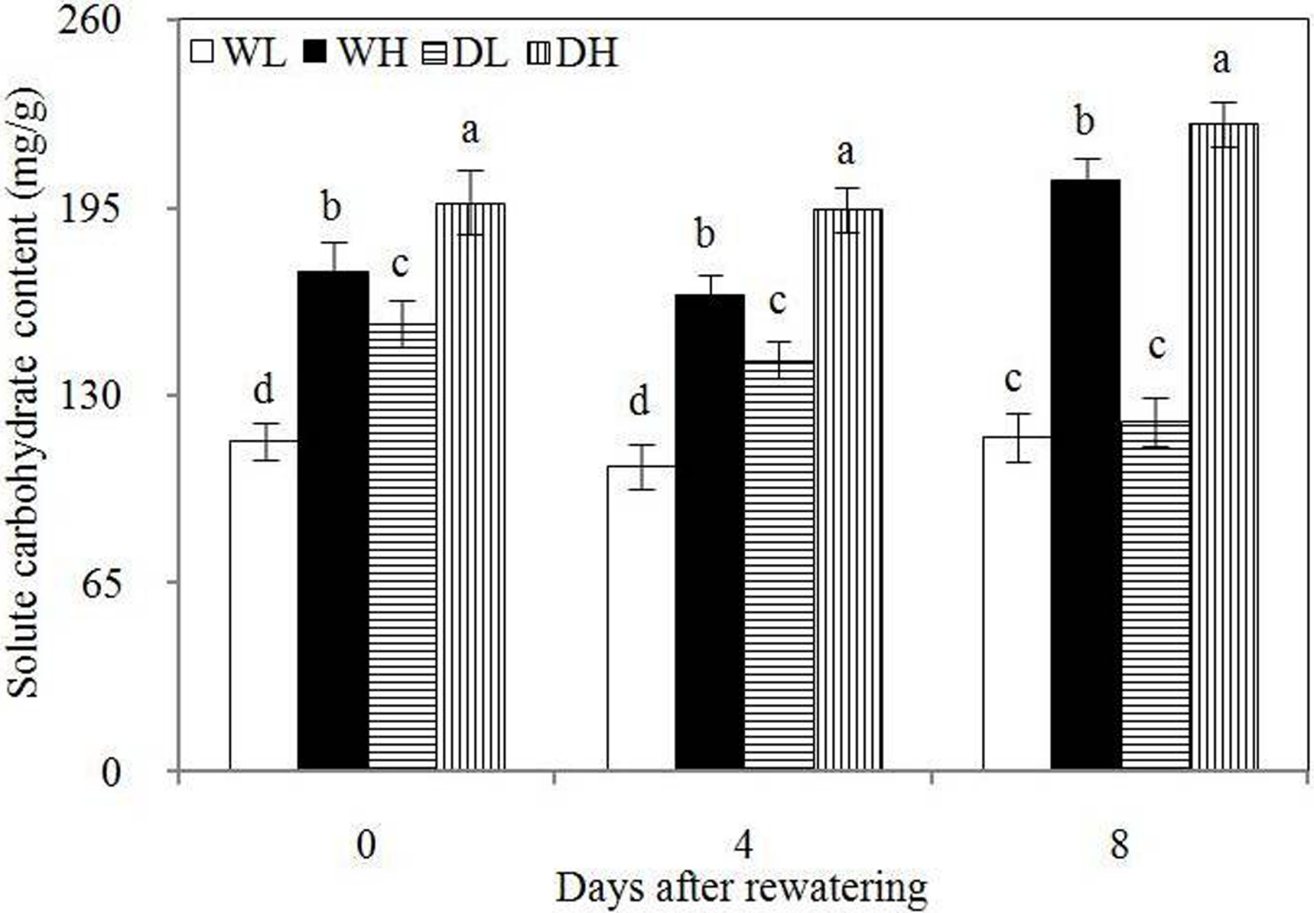
Soluble carbohydrate content in the different treatments. Error bars indicate± 1 standard deviation. For each number of days after rewatering, the different letters indicate significant differences at P ≤ 0.05.WL: wetness with low PPD; WH: wetness with high PPD; DL: rewatering with low PPD; DH: rewatering with high PPD.

As shown in Fig 4, DL and DH showed significantly higher soluble carbohydrate contents of roots compared with WL and WH before rewatering, respectively. Before rewatering and at 4 and 8 days after rewatering, WH and DH showed significantly higher root soluble carbohydrate content than WL and DL, respectively. Therefore, drought stress and high PPD could increase carbohydrate accumulation in roots. During the 8 day rewatering period, higher root soluble carbohydrate content was observed in DH than in WH, but this difference was observed in DL relative to WL only at 4 days after rewatering. These results showed that rewatering at low PPD could decrease the soluble carbohydrate content in roots over time but played a weak role in decreasing root soluble carbohydrate content under high PPD.

### Plant hormones

**Fig 5:**
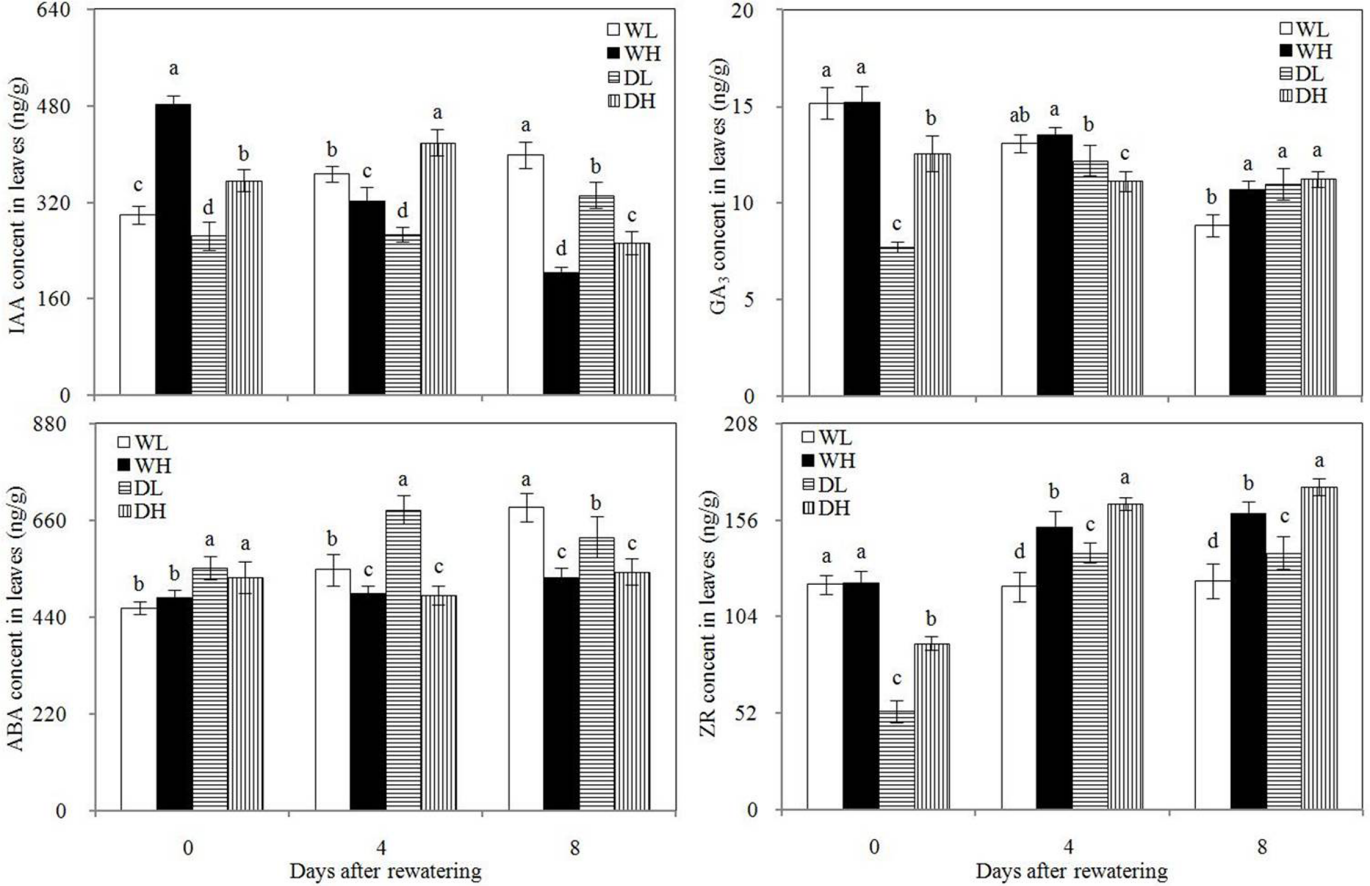
Leaf indole-3-acetic acid (IAA), gibberellic acid (GA_3_), abscisic acid (ABA), and zeatin riboside (ZR) contents in different treatment groups. Error bars indicate± 1 standard deviation. For each number of days after rewatering, the different letters indicate significant differences at P ≤ 0.05.WL: wetness with low PPD; WH: wetness with high PPD; DL: rewatering with low PPD; DH: rewatering with high PPD.

**Fig 6:**
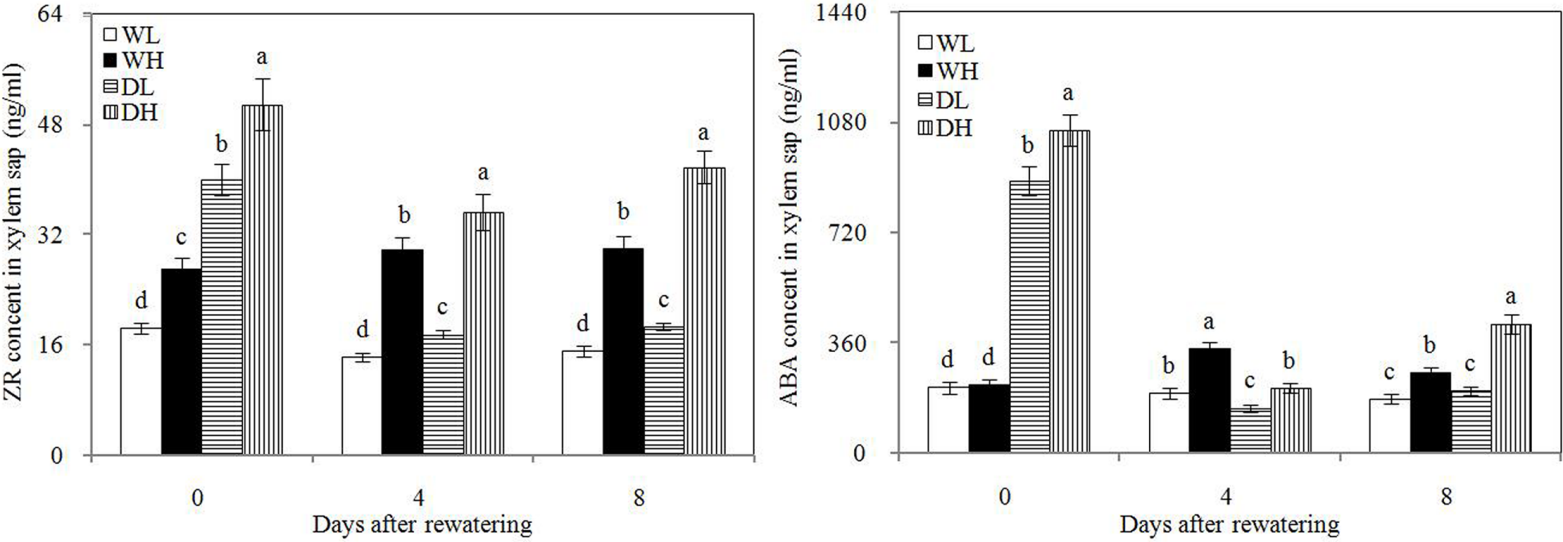
Zeatin riboside (ZR) and abscisic acid (ABA) contents of xylem sap in different treatment groups. Error bars indicate± 1 standard deviation. For each number of days after rewatering, the different letters indicate significant differences at P ≤ 0.05.WL: wetness with low PPD; WH: wetness with high PPD; DL: rewatering with low PPD; DH: rewatering with high PPD.

In Figs 5 and 6, before rewatering, WL and WH showed higher leaf IAA and GA_3_ contents and lower ABA contents in the leaves and xylem saps than DL and DH, respectively. However, these phenomena did not occur at 4 and 8 days after rewatering. Thus, drought stress decreases the IAA and GA_3_ contents and increased ABA content in the leaves and xylem saps, and post-drought rewatering altered this effect. PPD exerted minimal effect on the IAA and GA_3_ contents in leaves, but high PPD could decrease the ABA content in leaves and increase the ABA concentration in xylem saps. At 4 and 8 days after rewatering, WH and DH showed significantly higher leaf ABA content than WL and DL, respectively, and WL and DL presented higher xylem sap ABA concentrations than WH and DH, respectively.

**Fig 7:**
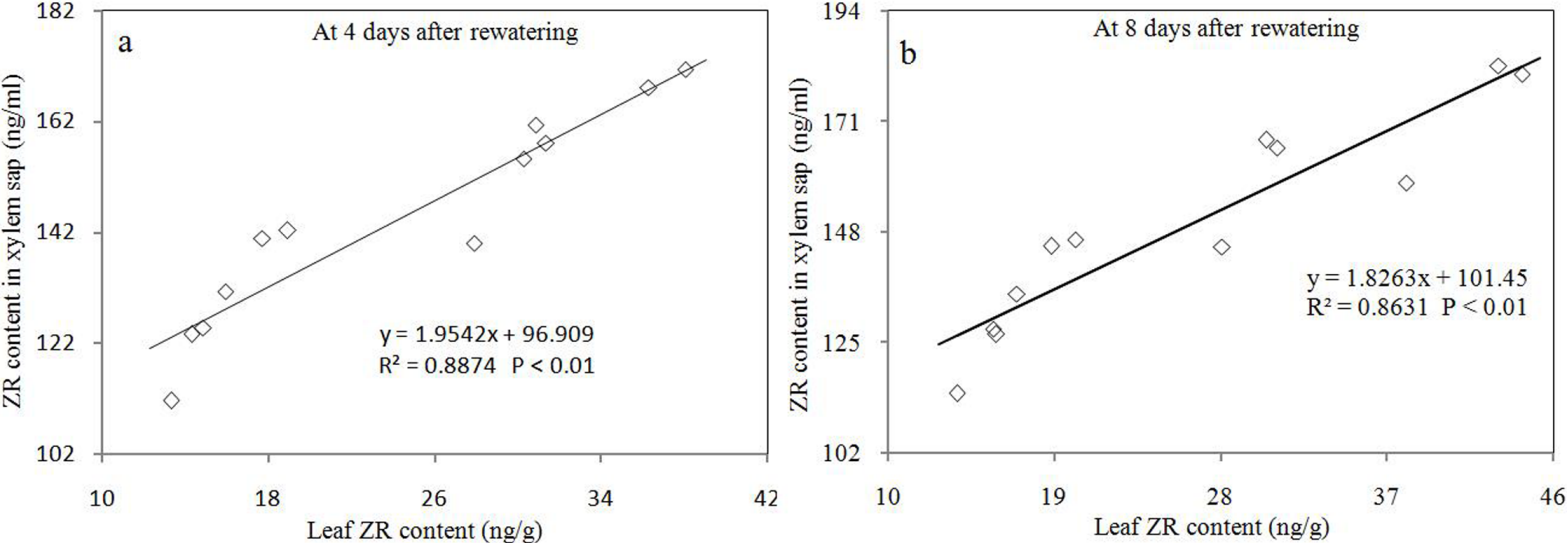
Relationship between ZR contents in leaves and xylem saps. In a and b, the regression lines are y = 1.9542x + 95.909 (R^2^ = 0.8874, n = 9, p < 0.01) and y = 1.8631x +101.45 (R^2^ = 0.8631, n = 9, p < 0.01), respectively.

Before rewatering, WL and WH showed higher leaf ZR content than DL and DH, respectively. ZR is the main form of cytokinin. This result showed that drought stress could decrease the leaf cytokinin content. DL and DH showed significantly higher leaf and xylem sap ZR contents at 4 and 8 days after rewatering compared with WL and WH, respectively. Therefore, rewatering increased the leaf cytokinin content and caused the root to secrete high concentrations of cytokinin. During drought stress and rewatering periods, WH and DH showed significantly higher leaf and xylem sap ZR contents than WL and DL, respectively, which showed that high PPD could increase these contents. A significant positive correlation was observed between leaf and xylem sap ZR concentrations at 4 and 8 days after rewatering (Fig 7).

## Discussion

### Photosynthesis and compensatory growth

In this study, compensatory growth during post-drought rewatering occurred in corn with high PPD during the 8 days of rewatering, but no compensatory growth was observed in corn with low PPD. Compensatory growth after post-drought rewatering essentially involves rapid growth and organic matter accumulation via leaf photosynthesis. The increase in the photosynthetic rate caused by rewatering was beneficial for compensatory growth of corn. During the rewatering period, the average photosynthetic rate of rewatering corns was 27.20% higher than wet corns under high PPD and only 15.31% higher than wet corns under low PPD. Thus, compensatory growth was more prone to occur under high PPD than under low PPD.

### Intraspecific competition and cytokinin

Cytokinin helps increase photosynthetic activity [25–28]. Similar phenomena were also observed in the current study. During the 8 days of rewatering, high leaf cytokinin concentration and photosynthetic rate were observed in rewatered corn plants compared with wet corn plants under high or low PPD, but the same horizontal concentrations for IAA, GA_3_ and ABA were observed in leaves between wet and rewatered corn plants. This finding was attributed to cytokinin regulating leaf stomatal conductance and leaf stomatal opening contributing to plant photosynthesis. Under high or low PPD, rewatered corn presented high stomatal conductance compared with wet corn. However, the large increase in the leaf photosynthetic rate caused by rewatering occurred in corn under high PPD compared with corn under low PPD. Therefore, leaf cytokinin was not definite influential factor of the photosynthetic rate under high PPD.

In the present study, high CV and *G* in high PPD corn exhibited more intense intraspecific competition and larger population size hierarchy compared with low PPD corn. Without self-thinning, growth resources of individual corn decreased with increasing PPD. Individual corn struggled to obtain growth resource with the result of intraspecific competition intensity enhancing. Simultaneously, the differences between individual sizes increased and some small-sized individuals occurred. The relatively small individuals required lesser growth resources, which might relieve competition intensity, thereby benefiting the population survival. By studying the population of spring wheat, Pan et al. [20] and Du et al. [19] both advocated that CV and *G* are indices that reflect population size hierarchy and intraspecific competition intensity. Similarly, under high PPD, wet treatment presented more intense intraspecific competition in the corn population compared with rewatering treatment, as shown by significantly higher CV and *G* in wet treatment than in rewatering treatment during the rewatering period. Drought stress caused relatively small-sized individual rewatered corn plants, which was confirmed by their low aboveground biomass before rewatering. Small-sized individuals required less growth resources than large individuals, thereby reducing intraspecific competition in the rewatered corn population under high PPD during the rewatering period.

In addition, wet corn plants showed lower photosynthetic rate than rewatered corn plants under high PPD. This finding may be mainly attributed to severe intraspecific competition, which negatively affected photosynthesis. Without self-thinning, individual corn struggled to obtain growth resources with increasing competition intensity, resulting in insufficient growth resource being supplied. Insufficient growth resource inevitably played a negative role in photosynthesis. This occurrence could alleviate competition intensity and retain population survival. Studies on cotton, corn, switchgrass, and spring rape populations all showed that plant population with high PPD presents low photosynthetic rate [29–33]. Therefore, photosynthetic activity must be restrained in wet populations under high PPD for drastic intraspecific competition. This phenomenon did not occur under low PPD due to weak intraspecific competition and close intraspecific competition intensity in the two populations. Thus, the interactions of leaf cytokinin and intraspecific competition caused compensatory growth to occur easily under high PPD.

### Roots and cytokinin

Wang et al. [13] reported that the leaf cytokinin concentration in rewatered corn plants is strongly influenced by roots. Rewatering plays a positive role on root function, thereby induced high leaf cytokinin concentration. This phenomenon also occurred in the present study, as demonstrated by the linear relationship between the leaf ZR content and the ZR content in xylem sap during rewatering. Plant roots are the primary sites of cytokinin synthesis, from which cytokinin is transported in the xylem sap to the leaves [34–36]. During rewatering, the high cytokinin concentration of xylem sap in rewatered corn under high or low PPD indicated that their roots were capable of secreting cytokinin and play a large role in the leaf cytokinin concentration. Consequently, high leaf cytokinin content was observed in rewatered corn under high or low PPD. Similarly, during rewatering, high cytokinin concentrations in xylem sap and leaves were observed in high PPD corn compared with low PPD corn under wet or rewatering treatment.

Sufficient supply of organic substances in roots can ensure active metabolism, resulting in the active division of meristematic zones and promotion of cytokinin synthesis. During rewatering, higher carbohydrate concentrations were observed in the roots in rewatering treatment compared with wet treatments; such concentrations benefited the excretion of cytokinin in the roots. High PPD corn also exhibited the advantage of increasing roots to secrete cytokinin because their roots possessed higher carbohydrate concentration than the roots of low PPD corn during rewatering period.

## Conclusion

In this study, drought stress inhibited corn seedling growth, whereas rewatering increased corn growth. Rewatering caused a larger increase in net photosynthetic rate under high PPD than under low PPD. During 8 days of rewatering, compensatory growth during post-drought rewatering easily occurred in high PPD corn, whereas no compensatory growth was observed in low PPD corn. Rewatering also increased ZR concentrations in leaves and xylem saps, and high leaf cytokinin concentration increased corn growth. Compared with wet corns, rewatered corn showed less intraspecific competition under high PPD, thereby benefiting increased growth and photosynthetic rate. The interaction of leaf cytokinin induced by roots and weak intraspecific competition caused compensatory growth to occur easily under relatively high PPD.

## Supporting information

S1 File Data files used in this study. This ZIP files contains all data used for this manuscript.
(RAR)
